# Biochemical Evidence for a Putative Inositol 1,3,4,5-Tetrakisphosphate Receptor in the Olfactory System of Atlantic Salmon (*Salmo salar*)

**DOI:** 10.1155/2013/460481

**Published:** 2013-03-11

**Authors:** Jiongdong Pang, Dennis E. Rhoads

**Affiliations:** ^1^Chemistry Department, Southern Connecticut State University, New Haven, CT 06515, USA; ^2^Department of Biochemistry, Microbiology and Molecular Genetics, University of Rhode Island, Kingston, RI 02881, USA; ^3^Department of Biology, Monmouth University, West Long Branch, NJ 07764-1898, USA

## Abstract

Olfactory receptor neurons in Atlantic salmon (*Salmo salar*) appear to use a phosphoinositide-directed phospholipase C (PLC) in odorant signal transduction. The consequences of odor-activated PLC depend on its product, inositol 1,4,5-trisphosphate (IP_3_). Therefore, a plasma membrane rich (PMR) fraction, previously characterized from salmon olfactory rosettes, was used to study binding sites for IP_3_ and its phosphorylation product, inositol 1,3,4,5-tetrakisphosphate (IP_4_). Binding sites for IP_3_ were present at the lower limit for detection in the PMR fraction but were abundant in a microsomal fraction. Binding sites for IP_4_ were abundant in the PMR fraction and thus colocalized in the same subcellular fraction with odorant receptors for amino acids and bile acids. Binding of IP_4_ was saturable and high affinity (*K*_*d*_ = 83 nM). The rank order for potency of inhibition of IP_4_ by other inositol polyphosphates (InsP*_x_*) followed the phosphorylation number with InsP_6_ > InsP_5_ > other InsP_4_ isomers > InsP_3_ isomers > InsP_2_ isomers, with the latter showing no activity. The consequences of PLC activity in this system may be dictated in part by a putative receptor for IP_4_.

## 1. Introduction

Adenylyl cyclase and cAMP appear to dominate odor signal transduction in mammals (for reviews, see [1–3]). Phosphoinositides may play a divergent role in olfaction, mediating inhibitory signaling through phosphoinositide-3-kinase [4] or excitatory signaling through phospholipase C [1, 5]. For fish, components of a phospholipase C-based olfactory signal transduction system have been characterized in catfish [6–13] and are seen in carp [14, 15], zebrafish [16], and Atlantic salmon [17, 18].

As potent olfactory stimuli for Atlantic salmon, amino acids and bile acids interact with distinct subclasses of olfactory receptors to begin the process of olfactory reception [18, 19]. The amino acid and bile acid receptors appear to be coupled through G proteins to the activation of phospholipase C (PLC) and the breakdown of phosphatidylinositol 4,5-bisphosphate (PIP_2_) to generate diacylglycerol (DAG) and inositol 1,4,5-trisphosphate (IP_3_) [17, 18]. Early biochemical data characterizing these as G protein-coupled receptors is now supported by molecular studies characterizing olfactory receptor gene sequences from Atlantic salmon [20–23]. Underscoring the importance of these receptors in salmon physiology, odorant receptor expression has been shown to change during the parr-smolt transformation, a period characterized by increased olfactory sensitivity and olfactory-based learning [24].

The significance of olfactory PLC activity resides in part with the location and characteristics of receptors for IP_3_. In most cells, IP_3_ receptors mediate the release of Ca^2+^ from internal stores in the endoplasmic reticulum (for review, see [25]). However, in association with PLC-based olfactory signal transduction, IP_3_ receptors have been found in olfactory cilia of catfish [6], carp [14], and lobster [26, 27]. From this position, IP_3_ may gate Ca^2+^ influx through the plasma membrane rather than the release from intracellular stores. Another important part of IP_3_ signaling in other systems has been its metabolism, including phosphorylation by a 3-kinase to generate the biologically active inositol 1,3,4,5-tetrakisphosphate (IP_4_) [28–30]. While IP_4_ continues to be studied in mammalian systems for roles as diverse as regulating nuclear calcium signaling [31], tyrosine kinase [32], and mitochondrial permeability and apoptosis [33, 34], Fadool and Ache [26] showed that olfactory receptor neurons of lobster express an IP_4_ receptor acting as a functional channel in the plasma membrane. In lobster, plasma membrane IP_3_ and IP_4_ receptors may interact reciprocally to regulate Ca^2+^ entry in olfactory neurons.

The goal of the present study was to characterize further the PLC-based olfactory signal transduction system of Atlantic salmon, beginning with the hypothesis that IP_3_ binding sites would colocalize with odor receptor binding sites in a plasma membrane rich fraction (PMR) that we characterized previously [17–20, 35]. Finding that binding of IP_3_ was marginal in this fraction, we proceeded to detect and characterize PMR binding sites for IP_4_ which may play a critical role in salmon olfactory transduction. Binding sites for IP_3_ were subsequently detected in the endoplasmic reticulum-rich microsomal fraction.

## 2. Materials and Methods

### 2.1. Isolation of the Plasma Membrane Rich (PMR) and Microsomal Fractions

Atlantic salmon (*Salmo salar*) were raised under conditions of simulated natural photoperiod and temperature in the aquaculture facility of University of Rhode Island. Using a modification of a method devised originally for rainbow trout by Cagan and Zeiger [36], a plasma membrane rich (PMR) fraction was obtained from the olfactory rosettes as described previously [19]. Rosettes were pooled from ten salmon for each analysis. The microsomal fraction was isolated from the olfactory rosettes using the method of Kalinoski et al. [6]. For comparative purposes, PMR fractions and microsomal fractions were also prepared from salmon brain and rat brain. Concentrations of proteins were determined by the method of Bradford (Bio-Rad Laboratories, Hercules, CA) with bovine serum albumin as a standard.

### 2.2. IP_3_ Binding

Binding of [^3^H]IP_3_ ([inositol-1-^3^H]; 21.0 Ci/mmol; New England Nuclear, Boston, MA) was measured using conditions described by Kalinoski et al. [6] except that microsomal fractions (100 *μ*g protein per assay) or PMR fractions (100–300 *μ*g protein per assay) were from salmon olfactory rosettes or from salmon or rat brain. Digitonin (50 *μ*g/mL) was added to permeabilize any membrane vesicles and insure that all binding sites are accessible [6]. The incubation buffer consisted of 110 mM KCl, 1 mM EGTA/0.2 mM CaCl_2_ (free Ca^2+^ concentration = 20 nM), and 10 mM HEPES, pH 7.4. Incubations were carried out for 30 min at 4°C. Separation of bound and free [^3^H]IP_3_ was achieved by rapidly filtering through Whatman GF/C filters and washing 3 times with assay buffer. Filters were extracted in scintillation cocktail for 4 hr, and the amount of associated radioactivity was determined by scintillation spectrometry. The amount of binding was determined in the absence (total binding) and presence (nonspecific binding) of excess (120 *μ*M) unlabeled InsP_3_. Two concentrations of [^3^H]IP_3_ (7 and 14 nM) were tested. The calculated difference between total and nonspecific binding was operationally defined as a specific binding.

### 2.3. IP_4_ Binding

The binding assay for [^3^H]IP_4_ ([Inositol-1-^3^H]; 21.0 Ci/mmol; New England Nuclear, Boston, MA) was performed under conditions identical to those described by Challiss et al. [37]. The assay buffer consisted of 25 mM CH_3_COONa, 25 mM KH_2_PO_4_, 5 mM NaHCO_3_, 1 mM EDTA, pH 5.0, and the indicated concentrations of [^3^H]IP_4_. Nonspecific binding was defined by the inclusion of 120 *μ*M unlabeled IP_4_. To characterize the binding specificity, competition assays were conducted with a minimum of three concentrations of other inositol polyphosphates (InsP_*x*_): InsP_6_, Ins(1,3,4,5,6)P_5_, Ins(3,4,5,6)P_4_, Ins(1,4,5,6)P_4_, Ins(1,3,4)P_3_, Ins(1,4,5)P_3_, Ins(1,4)P_2_, and Ins(4,5)P_2_ (all generously provided by Dr. Ching-Shih Chen, School of Pharmacy, University of Rhode Island). Reactions were initiated by the addition of PMR fraction (100 *μ*g protein), and samples were maintained at 4°C for 30 min with gentle rocking. Separation of bound and free [^3^H]IP_4_ was achieved by rapidly filtering through Whatman GF/C filters and washing 3 times with assay buffer. Filters were extracted in scintillation cocktail for 4 hr, and radioactivities were determined.

Binding assays for both [^3^H]IP_3_ and [^3^H]IP_4_ were based on conditions optimized by others ([6, 36], resp.). To rule out any effect of the different incubation conditions (most notably pH) on conclusions regarding binding of [^3^H]IP_3_ or [^3^H]IP_4_, each was tested at the conditions that had been optimized for the other. As expected, binding was negligible when measured at nonoptimal conditions.

## 3. Results

### 3.1. IP_3_ Binding

**Figure 1:**
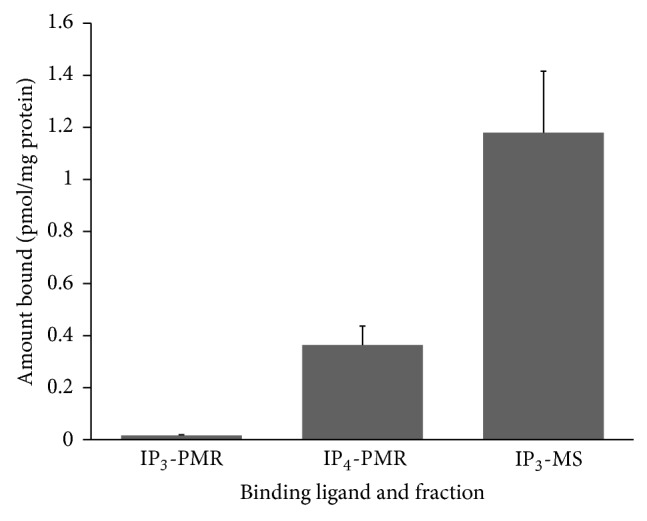
Initial screening of IP_3_ and IP_4_ binding sites in membrane fractions from salmon olfactory rosettes. The plasma membrane rich (PMR) fraction and a microsomal (MS) fraction were prepared from olfactory rosettes of Atlantic salmon. Specific binding of [^3^H]IP_3_ was determined in incubations with 14 nM [^3^H]IP_3_ in the presence and absence of excess unlabeled InsP_3_. Specific binding of IP_4_ to the PMR fraction is shown for comparison to IP_3_. The concentration of [^3^H]IP_4_ in the reaction mixture was also 14 nM.

At a radioligand concentration of 7 nM, no specific binding of IP_3_ was detectable with the olfactory PMR fraction. At 14 nM radioligand, IP_3_ binding to the olfactory PMR fraction was at the lower limit of detection in the assay (see data labeled IP_3_-PMR in Figure 1). Nonspecific binding accounted for almost 90% of the small amount of total binding of [^3^H]IP_3_ to the PMR fraction. Similar results were obtained with a salmon brain PMR fraction, analyzed as a negative control. The specific binding of [^3^H]IP_3_ corresponded to a maximum of 16 fmol bound per mg olfactory PMR protein and 10 fmol per mg salmon brain fraction.

In contrast, specific binding sites for [^3^H]IP_3_ were readily detected in a microsomal (MS) preparation from salmon olfactory rosettes (see data labeled IP_3_-MS in Figure 1). In this preparation, specific binding accounted for at least 75% of the total binding of [^3^H]IP_3_ and corresponded to 1.2 pmol IP_3_ bound per mg MS protein, a level nearly 100 times higher than the PMR fraction. This compares favorably to the level of IP_3_ binding measured in a rat brain microsomal fraction that was analyzed as a positive control.

### 3.2. IP_4_ Binding

While IP_3_ binding to the salmon olfactory PMR fraction was at the lower limit for detection in our assay, binding sites for IP_4_ were readily detected and were present at high density (see data labeled IP_4_-PMR in Figure 1). At comparable ligand concentration (14 nM), the olfactory PMR fraction supported binding of 364 fmol IP_4_ per mg protein (contrasted with 16 fmol IP_3_ per mg protein). Nonspecific binding represented less than 20% of total binding. In a single trial with the microsomal preparation from salmon olfactory rosettes, specific binding of [^3^H]IP_4_ was at the lower limit of detection (not shown). Thus, IP_4_ sites were readily detected in the PMR but not the microsomal fraction, a result opposite of that for IP_3_ binding.

**Figure 2:**
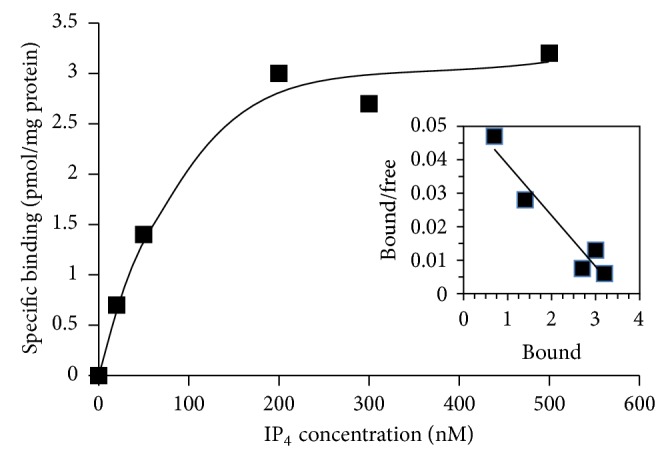
Saturation binding of [^3^H]IP_4_ to a plasma membrane rich fraction from salmon olfactory rosettes. Specific binding of IP_4_ was determined at each of the IP_4_ concentrations shown. Results are averaged from a single experiment performed in duplicate using olfactory rosettes from 10 Atlantic salmon and are representative of the results of three independent experiments. The inset shows a Scatchard analysis of the binding of IP4 to the plasma membrane rich fraction. Binding data as in Figure 2 was transformed to estimate the *K*_*d*_ and *B*_max_. The data fit a straight line (*R* ^2^ = 0.93) indicative of a single class of binding sites.

Experiments performed with increasing concentrations of [^3^H]IP_4_ demonstrated that specific binding was saturable (Figure 2). Scatchard analysis of the binding data (Figure 2, inset) yielded 83 nM for the *K*_*d*_ and 3811 fmol/mg protein for the *B*_max_ for IP_4_ binding to the olfactory PMR fraction.

**Figure 3:**
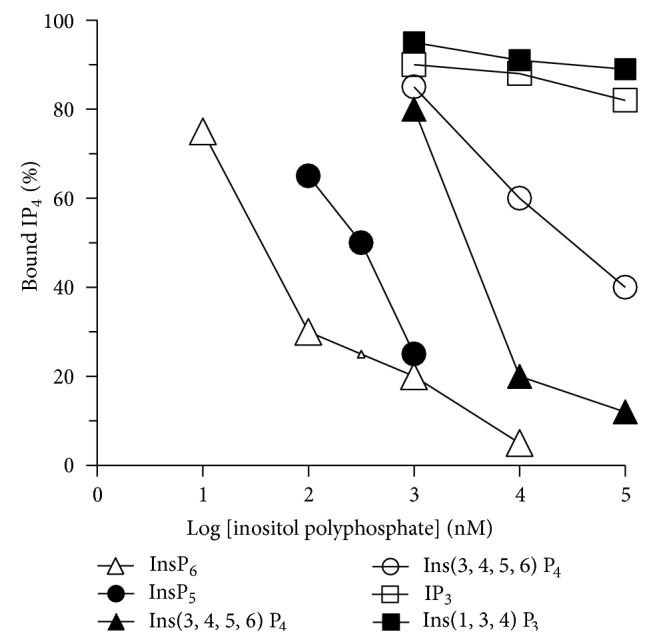
Inositol polyphosphate selectivity in competing for [^3^H]IP_4_ binding to a PMR fraction from salmon olfactory rosettes. The relative amount of specific binding of 14 nM [^3^H]IP_4_ was determined in the presence of at least three concentrations of different inositol polyphosphates (InsP_*x*_): InsP_6_ (open triangles), Ins(1,3,4,5,6)P_5_ (filled circles), Ins(3,4,5,6)P_4_ (filled triangles), Ins(1,4,5,6)P_4_ (open circles), Ins(1,3,4)P_3_ (open squares) and Ins(1,4,5)P_3_ (filled squares). The level of specific binding of [^3^H]IP_4_ in the absence of competitors was set at 100%.

To further characterize the specificity of IP_4_ binding to the olfactory PMR fraction, competition experiments were performed using 14 nM [^3^H]IP_4_ and various other inositol polyphosphates (InsP_*x*_) differing in degree and position of phosphorylation (Figure 3). If an analog competes with IP_4_, then binding will decrease as the concentration of the analog increased (Figure 3).

InsP_5_ and InsP_6_ showed reasonably potent inhibition of [^3^H]IP_4_ binding. Other IP_4_ analogs (Ins(3,4,5,6)P_4_ and Ins(1,4,5,6)P_4_) were intermediate in potency as inhibitors, while the IP_3_ analogs (Ins(1,3,4)P_3_ and Ins(1,4,5)P_3_) showed little or no activity. Similarly, Ins(1,4)P_2_ and Ins(4,5)P_2_, the dephosphorylation products formed from the inactivation of Ins(1,4,5)P_3_, had no inhibitory effect on [^3^H]IP_4_ binding when incubated at 10 *μ*M (data not included in Figure 3). From these competition assays, the effective concentration of analog giving 50% inhibition of [^3^H]IP_4_ binding (EC_50_) was estimated (Table 1). From this analysis, the rank order for potency of inhibition to [^3^H]IP_4_ binding was InsP_6_ > Ins(1,3,4,5,6)P_5_ > Ins(3,4,5,6)P_4_ > Ins(1,4,5,6)P_4_ > Ins(1,3,4)P_3_ = Ins(1,4,5)P_3_.

## 4. Discussion

Previous characterization of the PMR fraction showed high levels of the plasma membrane marker Na, K-ATPase and binding sites for amino acid [19, 20] and bile acid [18] odors. This fraction had minimal contamination with endoplasmic reticulum as suggested by the absence of thapsigargin-sensitive Ca^2+^-ATPase [35]. The low level of observed IP_3_ binding can also be considered as evidence of the lack of ER especially when compared to the microsomal fraction which is traditionally used as a source of endoplasmic reticulum and IP_3_ receptors [25]. A comparison of IP_3_ binding to the two subcellular fractions is consistent with the presence of IP_3_ receptors in endoplasmic reticulum rather than plasma membranes. This does not rule out the possibility that IP_3_ receptors would be detected at a higher level in isolated cilia [6] rather than the PMR fraction, but the low level of IP_3_ binding to the olfactory PMR fraction contrasts sharply with the high density of binding sites corresponding to odorant amino acid receptors [19, 20]. Clearly, IP_3_ receptors do not colocalize with odorant receptors in this fraction. Thus, our initial hypothesis that IP_3_ binding sites would be abundant in the PMR fraction from the olfactory rosettes of Atlantic salmon was not supported by this study.

In contrast, IP_4_ binding sites were abundant in this PMR fraction, which was previously shown to support odor-stimulated PLC activity [17, 18, 20]. Thus, it is an IP_4_ binding that colocalizes with odor receptors in the PMR fraction from salmon. Although the binding sites for IP_4_ appear in the PMR fraction with odor binding sites, we cannot confirm from this result alone that they appear together on the same membrane. In the only other olfactory system in which it has been characterized, IP_4_ gated a calcium channel in the lobster olfactory system [26]. If in salmon, the colocalization of odor and IP_4_ binding sites in the PMR fraction extends to a common membrane location, then an IP_4_ receptor could be an important downstream element in salmon olfactory transduction. The pH optimum and high affinity *K*_*d*_ value for IP_4_ binding are similar to what has been reported in mammalian brain, but the profile for the competition by other InsP_*x*_ is somewhat different [37]. The *B*_max_ for IP_4_ binding reflects a density of sites comparable to the density of IP_3_ binding sites in the olfactory plasma membrane of catfish (*B*_max_ = 17.6 pmol/mg protein from Kalinoski et al. [6]). The *K*_*d*_ value for IP_4_ binding is much lower (i.e., the affinity is much higher) than the *K*_*d*_ for IP_3_ binding sites in catfish (*K*_*d*_ = 1.1 *μ*M from Kalinoski et al. [6]), which is consistent with the lower level of IP_4_ produced relative to IP_3_ [38].

In essentially all animal cells, IP_3_ is metabolized in a bifurcate pathway that includes phosphorylation by a 3-kinase to produce IP_4_ [28, 39]. Higher-order inositol polyphosphates are also produced in cells along with an array of dephosphorylation products. We included many of these inositol polyphosphates in competition analyses to further characterize the olfactory IP_4_ binding site. Among the inositol polyphosphates tested, InsP_5_ and InsP_6_ showed reasonably potent inhibition of [^3^H]IP_4_ binding. These are formed by the sequential actions of specific kinases, are inhibitors of IP_4_ 3-phosphatase and IP_4_ 5-phosphatase [40], and are active in other cellular systems [41]. In contrast, Ins(1,3,4)P_3_, Ins(1,4)P_2_, and Ins(4,5)P_2_ showed little or no ability to interact with the IP_4_ site. This is not surprising because these are regarded as the products of inactivating phosphatases. Marginal inhibition of [^3^H]IP_4_ binding by IP_3_ (Ins(1,4,5)P_3_) confirmed the independence of the IP_4_ and IP_3_ binding sites in this system and supported the conclusions from direct measurements of [^3^H]IP_3_ binding at optimal pH that these sites are not present in the PMR fraction.

In summary, we found a unique IP_4_ binding site that colocalizes with odor receptors in a subcellular fraction derived from the olfactory system of Atlantic salmon. This is the first biochemical evidence of a putative membrane-bound IP_4_ receptor in a fish olfactory system. The exact plasma membrane location and the colocalization of odor receptors and putative IP_4_ receptors in the same plasma membrane remain to be shown. In the only other olfactory system in which it has been studied, electrophysiological studies have demonstrated that IP_4_ gates a calcium channel and helps regulate Ca^2+^ entry into lobster olfactory neurons [26], a similar role to that ascribed to IP_3_ in lobster [27], catfish [6], and carp [14]. This provides the only context with which to interpret the significance of finding IP_4_ binding sites in membranes of the salmon olfactory system and to begin to suggest that IP_4_ rather than (or in addition to) IP_3_ may be a key downstream element for olfactory signal transduction in Atlantic salmon.

## Figures and Tables

**Table 1:** EC_50_ values for inhibition of IP_4_ binding by inositol polyphosphates.

Inositol polyphosphate	EC_50_
InsP_6_	42.7 nM
Ins(1,3,4,5,6)P_5_	316 nM
Ins(3,4,5,6)P_4_	3.2 *μ*M
Ins(1,4,5,6)P_4_	31.6 *μ*M
Ins(1,4,5)P_3_	>100 *μ*M
Ins(1,3,4)P_3_	>100 *μ*M
Ins(1,4)P_2_	No inhibition at 10 *μ*M
Ins(4,5)P_2_	No inhibition at 10 *μ*M

Concentrations of the competing inositol polyphosphate (InsP_*x*_) effective in reducing [^3^H]IP_4 _binding to 50% of the specific binding (EC_50_) were derived from competition curves as shown in Figure 3. Incubations included 14 nM [^3^H]IP_4_.
